# Dexamethasone alleviates etomidate-induced myoclonus by reversing the inhibition of excitatory amino acid transporters

**DOI:** 10.3389/fnins.2024.1399653

**Published:** 2024-06-24

**Authors:** Yan Feng, Min Zhang, Shuai-ying Jia, Yan-xia Guo, Xue Jia

**Affiliations:** ^1^Department of Anesthesiology, Affiliated Hospital of North Sichuan Medical College, Nanchong, China; ^2^Department of Anesthesiology, Sichuan Integrative Medicine Hospital, Chengdu, China; ^3^Department of Anesthesiology, West China Hospital, Sichuan University, Chengdu, China

**Keywords:** etomidate, myoclonus, dexamethasone, EAATs, adrenal suppression

## Abstract

**Background:**

Etomidate can induce myoclonus with an incidence of 50 ~ 85% during anesthesia induction. Dexamethasone, as a long-acting synthetic glucocorticoid, has neuroprotective effects. However, the effects of dexamethasone on the etomidate-induced myoclonus remain uncertain.

**Methods:**

Adult male Sprague–Dawley rats were randomly assigned to receive etomidate (1.5 mg/kg) plus dexamethasone (4 mg/kg) (etomidate plus dexamethasone group) or etomidate (1.5 mg/kg) plus the same volume of normal saline (NS) (etomidate plus NS group). The mean behavioral scores, local field potentials and muscular tension were recorded to explore the effects of dexamethasone on etomidate-induced myoclonus. Liquid chromatography coupled with tandem mass spectrometric system (LC–MS/MS), quantitative real-time polymerase chain reaction (qRT-PCR), and western blotting were applied to analyze the levels of glutamate and *γ*-aminobutyric acid (GABA), the mRNA and protein expression of excitatory amino acid transporters (EAATs), and plasma corticosterone levels at different time points after anesthesia.

**Results:**

Compared with the etomidate plus NS treatment, the etomidate plus dexamethasone treatment significantly decreased the mean behavioral score at 1, 3, 4, and 5 min after administration; the peak power spectral density (PSD) (*p* = 0.0197) in the analysis of ripple waves; and the glutamate level (*p* = 0.0139) in the neocortex. However, compared with etomidate plus NS, etomidate plus dexamethasone increased the expression of the neocortical proteins of EAAT1 (*p* = 0.0207) and EAAT2 (*p* = 0.0022) and aggravated the inhibition of corticosterone at 4 h (*p* = 0.0019), 5 h (*p* = 0.0041), and 6 h (*p* = 0.0009) after administration.

**Conclusion:**

Dexamethasone can attenuate the myoclonus, inhibit the glutamate accumulation, and reverse the suppression of EAATs in the neocortex induced by etomidate following myoclonus, while conversely aggravating etomidate-induced adrenal suppression.

## Introduction

Etomidate, an imidazole-based agonist of *γ*-aminobutyric acid type A (GABA_A_) receptor, has a short-lasting and rapid onset of action, is frequently used in clinical practice as a sedative-hypnotic agent for the attractive characteristics, such as stable haemodynamics and limited respiratory depression (He et al., 2014). Hypnosis can last for 5–10 min after the intravenous injection at the dose of 0.3 mg/kg. Nevertheless, intravenous injection of etomidate usually results in myoclonus, a highly reported incidence of 50 ~ 85% with a sudden and brief involuntary twitching or jerking of a muscle or group of muscles that can last from seconds to minutes during anesthesia induction (Kojovic et al., 2011). Etomidate-related myoclonus may develop into some serious complications, such as accidental dislocation of the intravenous tube and monitoring devices, reflux aspiration and the increased potassium blood levels, which has become an urgent clinical problem to be solved (Hua et al., 2019). Sedative or analgesic drugs, including benzodiazepines, opioids, *N*-methyl-d-aspartate receptor (NMDAR) antagonists or α2 receptor agonists, can prevent the etomidate-induced myoclonus, but these drugs may increase the risk of respiratory depression, arrhythmia or hypotension (Zhang et al., 2022).

Dexamethasone is a long-acting synthetic glucocorticoid with a plasma half-life of 4–5 h (Tanaka et al., 1997). Dexamethasone has several therapeutic effects, such as inhibiting the systemic inflammatory response; decreasing the incidence of postoperative pain, nausea, and vomiting (Busti et al., 2005; De Oliveira et al., 2011; Waldron et al., 2013; Pehora et al., 2017; Jensen et al., 2020), and is commonly used during the perioperative period. Dexamethasone also has potential neuroprotective effects. Sinha et al. (2021) reported that dexamethasone can ameliorate the neuroinflammation and seizure susceptibility in the Lafora disease animals by reducing the glial activation. Tan et al. (2021) found that dexamethasone improved short-term and long-term cognitive impairment in adult rats induced by sevoflurane. In addition, Barna et al. (2020) demonstrated that dexamethasone could protect the blood–brain barrier from damage and reduce the severity of seizures in pilocarpine-induced status epilepticus. However, the effect of dexamethasone on etomidate-induced myoclonus has not been determined. As we known, etomidate-induced myoclonus involves neocortical glutamate accumulation and NMDAR modulation, and suppresses the protein expression of excitatory amino acid transporters (EAATs) in the motor cortex (Feng et al., 2023). EAAT1 and EAAT2 are predominantly expressed in astroglial cells and are mainly responsible for the glutamate uptake (Schneider et al., 2021). However, the effect of dexamethasone on glutamate levels and the protein expressions of EAATs following etomidate-induced myoclonus has not been determined.

Moreover, etomidate inhibits the steroidogenesis occurring at the level of the adrenal gland, the inhibition induced by etomidate depends on the plasma concentration of etomidate and physical state of the patients (Feng et al., 2024). A single intravenous injection of etomidate (0.3 mg/kg) may result in adrenal insufficiency (AI) in critically ill patients (Molenaar et al., 2012), but the same administration of etomidate in healthy patients has the potential to decrease the cortisol level and this reduction resulted in stress response inhibition without adverse effects (Schenarts et al., 2001). Glucocorticoid supplementation for prevention of etomidate-induced AI remains controversial according to the previous studies (Dellinger et al., 2008; Payen et al., 2012). The effects of dexamethasone on etomidate-related adrenal suppression have rarely been reported. We thus tested the primary hypotheses that dexamethasone can attenuate etomidate-induced myoclonus by inhibiting the neocortical glutamate accumulation and reversing the suppression of EAATs, and may prevent the etomidate-induced adrenal suppression as a glucocorticoid supplement.

## Materials and methods

### Animals

All experimental procedures were approved by the Animal Ethics Committee of West China Hospital, Sichuan University (ethical approval number: 20211423A) and conducted in strict accordance with the Guide for the Care and Use of Laboratory Animals published by the United States National Institutes of Health (National Research Council (US) Committee for the Update of the Guide for the Care and Use of Laboratory Animals, 2011). Adult male rats (6–8 weeks old) were used for the experiments. All rats were housed at 25 ± 1°C with 60% humidity in the Animal Experimental Center of Sichuan University (Chengdu, China) on a 12-h light/dark cycle (lights on at 7:00 a.m.) and provided with water and food *ad libitum*. The rats were habituated to the experimental environment for 1 week before testing. The rats were numbered randomly and assigned to experimental groups, and tested in sequential order.

### Quantification of etomidate-induced myoclonus

Adult male rats were randomly divided into two groups (*n* = 10/group): those that received intravenous doses of etomidate (1.5 mg/kg) [twice the 50% effective dose {ED50} (Wang et al., 2017)] (YT200712, Nhwa Pharma. Corporation, Xuzhou, China) plus a potential anti-epileptic dose of dexamethasone (4 mg/kg) (Sinha et al., 2021) (22,308,031, Zhaohui Pharma. Corporation, Shanghai, China), those who received etomidate (1.5 mg/kg) plus the same volume of normal saline (NS). Dexamethasone or NS was given after the etomidate administration. The rats were placed in transparent cages individually and observed at 0, 1, 2, 3, 4, and 5 min after anesthesia to record the mean behavioral score. Behavioral scores were determined according to the modified Racine Scale (Yuan et al., 2020), as follows: 0, no response; 1, ear and facial twitching; 2, myoclonic jerks without rearing; 3, myoclonic jerks and rearing; 4, turning over onto the side and tonic–clonic seizures; and stage 5, turning over onto the back and generalized tonic–clonic seizures.

### Local field potential recordings and muscle tension monitoring

Local field potentials (LFPs) were explored using an Ag/AgCl reference electrode in a glass capillary microelectrode (1- to 2-μm diameter tip) filled with 150 mM NaCl. The tip of the glass capillary microelectrode was implanted into the right neocortex (from the bregma: 2.0 mm anteroposterior; 2.7 mm mediolateral; 1.8 mm dorso-ventral) according to the hindlimb motor cortex map (Neafsey et al., 1986) and the Paxinos and Watson atlas (Paxinos and Watson, 1986). The end of the glass capillary micro-electrode, attached to a drug delivery system, which was administered through a microsyringe and connected to a microelectrode amplifier (ME-1, Chengdu Techman Software Co., Ltd., Chengdu, China). The recorded signal was subsequently analyzed with BL420N Biological Signal Acquisition and Analysis System software (Chengdu Techman Software Co., Ltd., Chengdu, China). LFPs were digitized at 2000 Hz and filtered at 0.5–500 Hz (raw data), and ripple signals (80–250 Hz). Notch filtering at 50 Hz was applied to remove noise. A calibration-free biotension sensor (FT-102) was used to monitor the muscular tension. The sensor was fixed to a holder to ensure that the direction of the force was perpendicular to the spring plate of the force and that the probe was implanted into the left gastrocnemius muscle. The signal was also recorded through the BL420N instrument. The time-frequency analysis of the raw LFPs, ripple waves, the peak muscular tension amplitude, and frequency were recorded to verify the origination of etomidate-induced myoclonus in adult male rats when anesthetized intravenously with etomidate (1.5 mg/kg for induction, 4 mg·kg^−1^·h^−1^ for maintenance), 4 mg/kg dexamethasone administered after induction (*n* = 10), or etomidate (1.5 mg/kg for induction, 4 mg·kg^−1^·h^−1^ for maintenance) plus the same quantity of normal saline (NS) (*n* = 10).

### Levels of glutamate and GABA measurement

The neocortical tissues (Santos et al., 2011) of adult male rats (*n* = 10/group) were extracted after intravenous administration of etomidate (1.5 mg/kg) plus dexamethasone (4 mg/kg), or normal saline for 5 min. Liquid chromatography coupled with tandem mass spectrometric system (LC–MS/MS) consisted of an Agilent 6,460 triple quadrupole mass spectrometer equipped with an electrospray ionization source (Agilent Technologies, CA), and the ion spray (IS) method was used to determine the glutamate and *γ*-aminobutyric acid (GABA) levels. The following parameters were used: column temperature, 35°C, the dry gas flow rate of 5.0 L/min; dry gas temperature, 350°C, sheath gas flow rate of 11.0 L/min; sheath gas heater temperature of 350°C, nebulizer pressure of 45 psi, capillary voltage of 3,500 V; and auxiliary voltage of 500 V. The data was analyzed using Mass Hunter software (Build 4.0.479.0, Agilent Technologies) (Feng et al., 2023).

### Quantitative real-time polymerase chain reaction (qRT-PCR) analysis of EAATs

After intravenous anesthesia with etomidate plus NS or etomidate plus dexamethasone for 5 min, 6 rats in the each group were sacrificed by decapitation. The neocortical tissues of 6 rats in each group were collected in frozen pipes and stored at −80°C for determination of the mRNA concentration of EAATs. Total RNA was extracted from the neocortex of each rat was extracted with TRIzol reagent (TaKaRa, Otsu, Shiga, Japan, #9109) on a clean bench (SW-CJ-1D, China) at a low temperature. The RNA from each sample was used to synthesize cDNA using a High-Capacity cDNA Reverse Transcription Kit (Toyobo, Tokyo, Japan, #FSQ-201) with the Automatic Medical PCR Analysis System (SCILOGEX, Shanghai, China). The sequences of the primers used for EAAT1 (5’-TCAGAACATCACCAAGGAGGA-3′ and 5’-TACGGTCGGAGGGCAAA-3′, primer5, NM_001289942.1); the sequences of the primers used for EAAT2 (5’-AGCCAAAGCACCGAAACC-3′ and 5’-AAGCAGCCCGCCACATA-3′, primer5, NM_017215.3); and the sequences of the primers used for GAPDH (5’-GGTGAAGGTCGGTGTGAACG-3′ and 5’-CTCGCTCCTGGAAGATGGTG-3′, primer5, NM_017008.4). The total RNA concentration was determined using an ultramicro spectrophotometer (Scandrop 100, China). qRT-PCR was performed on an Automatic Medical PCR Analysis System with SYBR^®^ Green Real-time PCR Master Mix in a final volume of 20 μL, with the following thermal cycling procedure: 95°C for 2 min, followed by 40 cycles of 95°C for 10 s, 58°C for 30 s, and 95°C for 15 s. A GAPDH positive control without a template was included for each amplification. The above real-time fluorescence quantification was repeated three times to obtain three *C*t values, and the average *C*t value was calculated. The data module was derived by the PCR software system, and the expression of EAAT1 and EAAT2 mRNA was quantitatively analyzed according to the RQ = 2^−ΔΔCT^ method (Feng et al., 2022).

### Western blot analysis of EAATs

Adult male rats (*n* = 6/group) were divided into two groups: the etomidate plus dexamethasone group and the etomidate plus NS group. The brains of the rats were immediately removed at 5 min after the administration of dexamethasone or NS and placed on ice. Proteins were extracted from neocortical tissues using radioimmunoprecipitation assay buffer (#P0013B, Beyotime, Shanghai, China) supplemented with a protease inhibitor cocktail (Roche Diagnostics GmbH, Mannheim, Germany). Standard protein electrophoresis was performed on 8% Tris-glycine polyacrylamide gels with loading buffer (#P0015L, Beyotime, China) and Multicolour Prestained Protein Ladder (WJ103, Epizyme, China). Wet transfer was performed with the Trans-Blot SD system (Bio-Rad). The blots were blocked with 5% non-fat dry milk (Bio-Rad), cut, and incubated with the following primary antibodies at 4°C overnight: EAAT1, Cell Signaling Technology, Danvers, MA, United States; #5684, 1:1,000 dilution; and EAAT2, Proteintech, #22515-1-AP, 1:1,000 dilution. The blots were then incubated with a horseradish peroxidase (HRP)-conjugated goat anti-rabbit secondary antibody (1:5000, #ZB2301 ZSGB-BIO, China) for 2 h at room temperature, The band densities of EAAT1 and EAAT2 were normalized to the band density of the loading control *α*-tubulin using ImageJ software.

### Sample preparation and analysis of corticosterone

Blood samples were collected into heparinized tubes at 0.5 h before as well as 1, 2, 3, 4, 5, and 6 h after administration of test drugs (etomidate plus dexamethasone and etomidate plus NS, *n* = 10/group). 50 μL of sample was added into a polypropylene tube and 150 μL of acetonitrile solution that contained corticosterone-d8 (20 ng/mL; internal standard, IS) was added to deproteinize the serum for the corticosterone measurement. After centrifugation (10,000 *g*, 10 min, 4°C), the supernatant was analyzed via LC–MS/MS system consisted of an Agilent 6,460 triple quadrupole mass spectrometer equipped with an electrospray ionization source (Agilent Technologies, CA, United States). Chromatographic separation was carried out using a Waters symmetry C18 column (3 mm × 100 mm, 3.5 μm) at 30°C. The mobile phase consisted of 0.1% formic acid (A) and acetonitrile (B) in a gradient elution process: 0 min (A at 60%), 1 min to 1.4 min (A at 40%), and 4.5 min to 4.6 min (A at 10%) at a flow rate of 0.3 mL/min. Mass spectrometry was performed in positive ionization mode: sheath gas flow rate, 11.0 L/min; sheath gas heater temperature, 300°C; nebulizer pressure, 45 psi; and capillary voltage, 3,500 V. MassHunter software (B.04.00 Build 4.0.479.0; Agilent Technologies, CA, United States) was used to analyze the data (Chang et al., 2022).

### Statistical analysis

Parametric data were reported as the means and standard deviations, and non-parametric data were reported as percentiles. A normal distribution was confirmed using the Shapiro–Wilk test. The mean behavioral scores and concentrations of serum corticosterone at different time points were compared between the two groups by a linear mixed-model analysis for repeated-measure, and a *post hoc* Bonferroni correction test was performed to adjust *p-*values for multiple comparisons. LFP and muscle tension recordings, glutamate and GABA levels, and mRNA and protein expression of EAATs, were compared via two-sample *t-*tests for normally distributed data or two-tailed Wilcoxon signed rank tests for non-normally distributed data.

The sample size was determined by using G*Power 3 (Faul et al., 2007) with the power (1-*β*) set at 0.85, and *α* = 0.05, which indicated that *n* = 10/group for the quantification of etomidate-induced myoclonus, *n* = 10/group for the recording of local field potentials and muscular tension, *n* = 10/group for the measurement of glutamate and GABA, *n* = 10/group for the analysis of corticosterone, *n* = 6/group for the qRT-PCR analysis and *n* = 6/group for the western blot analysis. Statistical analyses were performed using Prism 9.0.0 software (GraphPad Software, San Diego, CA, United States) and SPSS software, version 19.0 (IBM SPSS Statistics, United States). *p <* 0.05 was considered to indicate statistical significance.

## Results

### Dexamethasone decreased behavioral score in etomidate-induced myoclonus

**Figure 1:**
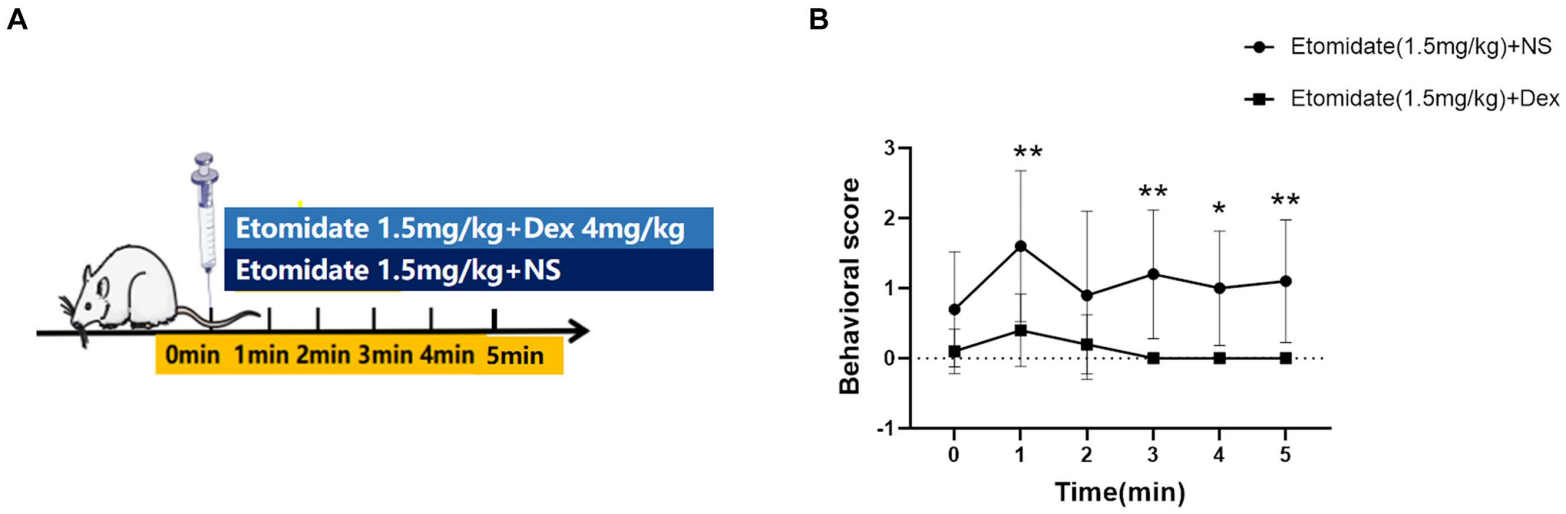
Effects of dexamethasone on the quantification of etomidate-induced myoclonus. **(A)** Adult male rats were anesthetized with etomidate (1.5 mg/kg) plus dexamethasone (4 mg/kg) and etomidate (1.5 mg/kg) plus same volume of normal saline (NS) (*n* = 10 rats/group). **(B)** The trend of behavioral score at different time points in the etomidate plus dexamethasone group and etomidate plus NS group. ^*^p < 0.05, ^**^p < 0.01, by a linear mixed-model analysis for repeated-measures and a post-hoc Bonferroni correction test for multiple comparisons. Dex, dexamethasone; NS, normal saline.

Treatment with etomidate plus dexamethasone decreased the mean behavioral score at 1 (mean difference [MD]: 1.20, 95% confidence interval [CI], 0.35–2.05; *p* = 0.0016); 3 (MD: 1.20, 95% CI, 0.35–2.05; *p* = 0.0016); 4 (MD: 1.00, 95% CI, 0.15–1.85; *p* = 0.0130); 5 (MD: 1.10, 95% CI, 0.25–1.95; *p* = 0.0047) min after administration compared to treatment with etomidate plus NS; but treatment with etomidate plus dexamethasone did not affect the mean behavioral score at 0 (MD: 0.60, 95% CI, −0.25–1.45; *p* = 0.3203) or 2 (MD: 0.70, 95% CI, −0.15–1.55; *p* = 0.1677) min after administration compared to treatment with etomidate plus NS, as determined by the linear mixed-model analysis and the *post hoc* Bonferroni correction test for multiple comparisons (Figure 1).

### Electrophysiological and muscular tension characteristics *in vivo*

**Figure 2:**
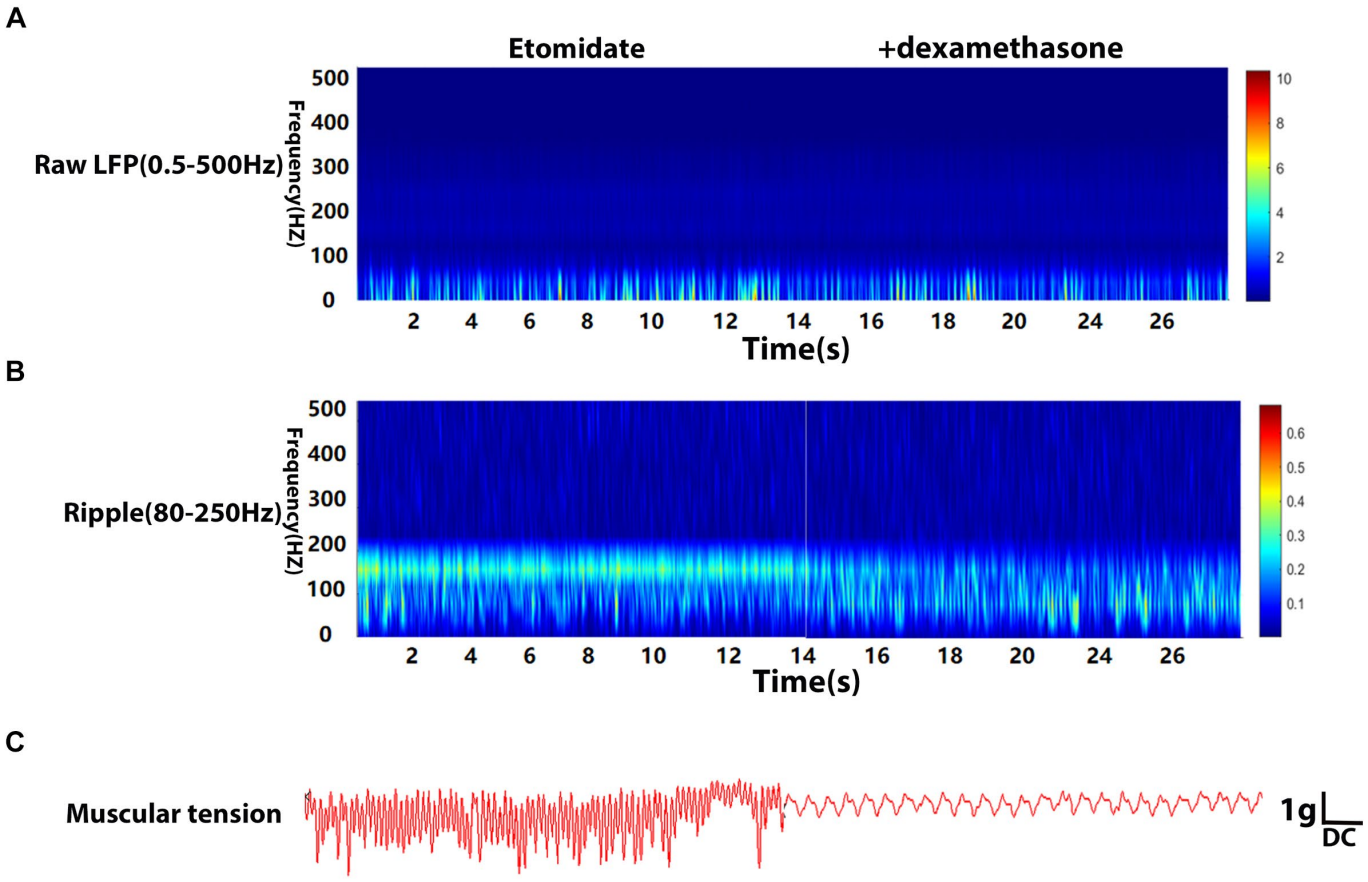
Electrophysiological and muscular tension characteristics in neocortex under etomidate + dexamethasone anesthesia. **(A)** Power spectrogram of raw LFP (0.5–500 Hz) recorded in neocortex for etomidate plus dexamethasone (*n* = 10 rats/group). **(B)** Power spectrogram of Ripple (80–250 Hz) in neocortex for etomidate plus dexamethasone. **(C)** Representative traces of right gastrocnemius muscle tension monitoring when the microelectrode was inserted into in neocortex for etomidate plus dexamethasone. A two-tailed *t-*test or two-tailed Wilcoxon signed rank test was applied to analyze difference.

**Figure 3:**
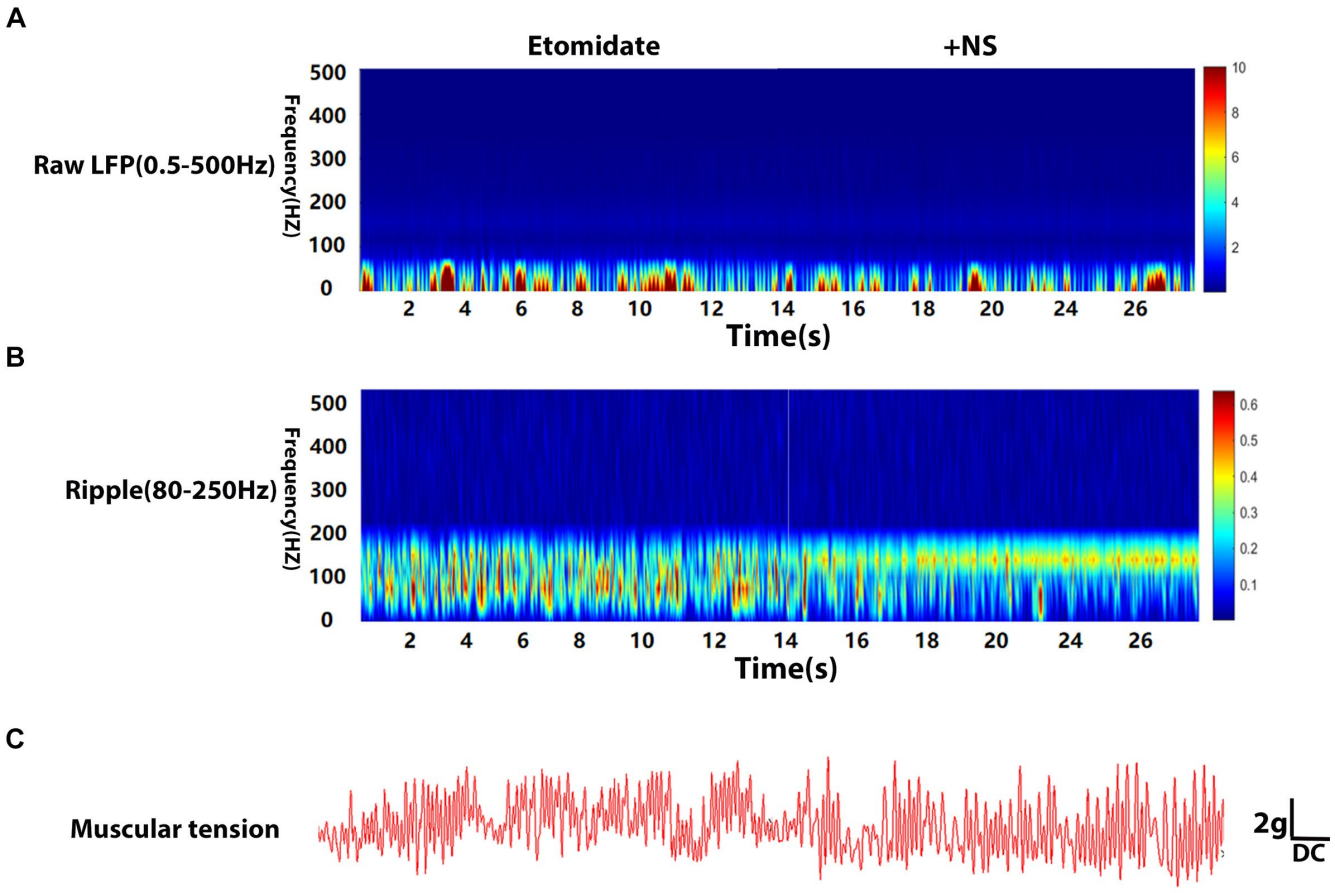
Electrophysiological and muscular tension characteristics in neocortex under etomidate + NS anesthesia. **(A)** Power spectrogram of raw LFP (0.5–500 Hz) recorded in neocortex for etomidate plus NS (*n* = 10 rats/group). **(B)** Power spectrogram of Ripple (80–250 Hz) in neocortex for etomidate plus NS. **(C)** Representative traces of right gastrocnemius muscle tension monitoring when the microelectrode was inserted into in neocortex for etomidate plus NS. A two-tailed *t-*test or two-tailed Wilcoxon signed rank test was applied to analyze difference.

The LFPs were recorded using deep electrodes implanted into the neocortex, and the results are listed in the Table 1. In the etomidate plus dexamethasone group, although etomidate plus dexamethasone did not affect the peak PSD (3.09 × 10^−4^ ± 4.13 × 10^−4^ vs. 2.74 × 10^−4^ ± 2.61 × 10^−4^ mV^2^/Hz, *p* = 0.8457) or frequency (13.00 ± 14.94 vs. 12.00 ± 10.33 Hz, *p* = 0.7500) in the analysis of raw LFPs (Figure 2A), and the peak frequency (98.00 ± 45.66 vs. 96.00 ± 38.64 Hz, *p* = 0.9688) of ripple waves, while significantly decreased the peak PSD (1.14 × 10^−6^ ± 8.09 × 10^−7^ vs. 2.76 × 10^−6^ ± 2.69 × 10^−6^ mV^2^/Hz, *p* = 0.0371) in the analysis of ripple waves, compared to the etomidate alone (Figure 2B). For the muscular tension, compared with etomidate alone, etomidate plus dexamethasone decreased the peak muscular tension amplitude (1.89 ± 1.34 vs. 6.68 ± 1.65 g, *p* = 0.0020) or frequency (0.93 ± 0.85 vs. 3.56 ± 3.32 Hz, *p* = 0.0137) compared to the etomidate alone (Figure 2C), as determined by two-sample paired *t-*test or two-tailed Wilcoxon signed rank test. In addition, in the etomidate plus NS group, etomidate plus NS did not affect the peak power spectral density (PSD) (6.02 × 10^−4^ ± 4.66 × 10^−4^ vs. 3.46 × 10^−4^ ± 3.92 × 10^−4^ mV^2^/Hz, *p* = 0.1602) or frequency (12.00 ± 13.17 vs. 15.00 ± 13.54 Hz, *p* = 0.5625) in the analysis of raw LFPs (0.5–500 Hz) when compared to the etomidate alone (Figure 3A). Regarding ripple waves (80–250 Hz), etomidate plus NS also affected neither the peak PSD (1.78 × 10^−6^ ± 1.51 × 10^−6^ vs. 2.29 × 10^−6^ ± 1.32 × 10^−6^ mV^2^/Hz, *p* = 0.3832) nor frequency (98.00 ± 45.66 vs. 112.00 ± 44.42 Hz, *p* = 0.6563) when compared to the etomidate alone (Figure 3B). For the muscular tension, etomidate plus NS also did not affect the peak muscular tension amplitude (7.04 ± 2.79 vs. 6.66 ± 2.86 g, *p* = 0.6388) or frequency (2.67 ± 1.87 vs. 2.36 ± 1.79 Hz, *p* = 0.9219) compared to the etomidate alone (Figure 3C), as determined by two-sample paired *t-*test or two-tailed Wilcoxon signed rank test.

**Table 1:** The characteristics of electrophysiological and muscular tension.

Location	Wave band	Iterms	Group			
			Etomidate + Dex group		Etomidate + NS group	
			Etomidate (baseline 1)	Etomidate + Dex	Etomidate (baseline 2)	Etomidate + NS
Neocortex	Raw data (0.5–500 Hz)	Peak power spectral density (mV^2^/Hz)	2.74 × 10^−4^ ± 2.61 × 10^−4^	3.09 × 10^−4^ ± 4.13 × 10^−4^	3.46 × 10^−4^ ± 3.92 × 10^−4^	6.02 × 10^−4^ ± 4.66 × 10^−4^
		Peak frequency (Hz)	12.00 ± 10.33	11.00 ± 13.70	15.00 ± 13.54	12.00 ± 13.17
	Ripple (80–250 Hz)	Peak power spectral density (mV^2^/Hz)	2.76 × 10^−6^ ± 2.69 × 10^−6^	1.14 × 10^−6^ ± 8.09 × 10^−7*^	2.29 × 10^−6^ ± 1.32 × 10^−6^	1.78 × 10^−6^ ± 1.51 × 10^−6^
		Peak frequency (Hz)	96.00 ± 38.64	98.00 ± 45.66	112.00 ± 44.42	98.00 ± 45.66
	Muscular tension	Peak amplitude (g)	6.68 ± 1.65	1.89 ± 1.34^*^	6.66 ± 2.86	7.04 ± 2.79
		Peak frequency (Hz)	3.56 ± 3.32	0.93 ± 0.85^*^	2.36 ± 1.79	2.67 ± 1.87

^*^P < 0.05; compared to Etomidate (baseline 1). Dex, dexamethasone; NS, normal saline.

### Dexamethasone inhibited the etomidate-induced neocortical glutamate accumulation

**Figure 4:**
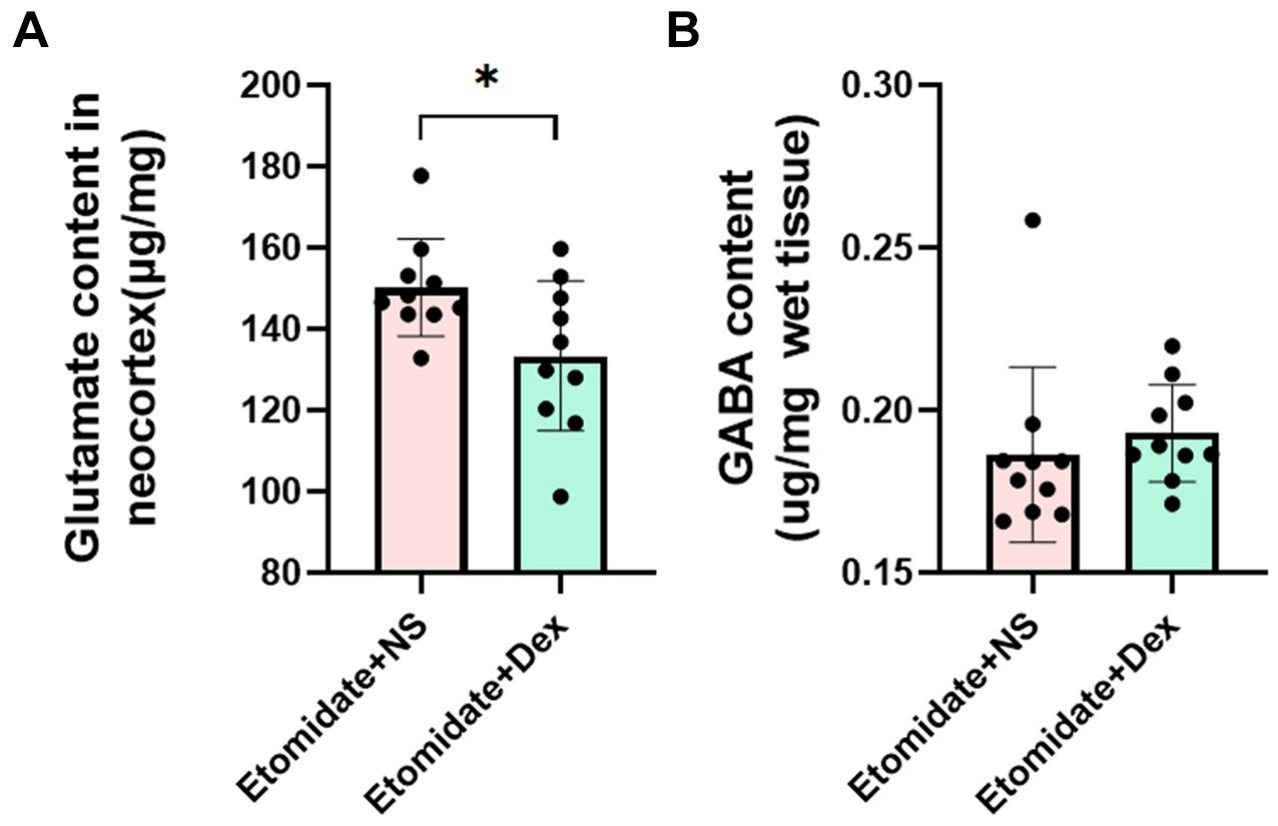
Quantification of glutamate and *γ*-aminobutyric acid (GABA) measurement. **(A)** Glutamate content in neocortex in the etomidate + dexamethasone group and etomidate + NS group (*n* = 10 rats/group). **(B)** GABA content in neocortex in the etomidate + dexamethasone group and etomidate + NS group (*n* = 10 rats/group). ^*^p < 0.05, by a two-tailed paired *t-*test or two-tailed Wilcoxon signed rank test. Dex, dexamethasone; NS, normal saline.

In the neocortex, compared to etomidate plus NS, etomidate plus dexamethasone significantly decreased the glutamate levels (1.502 ± 0.119 vs. 1.334 ± 0.184 μg/mg, *p* = 0.0139) (Figure 4A), but did not affect the GABA levels (0.193 ± 0.015 vs. 0.186 ± 0.027 μg/mg, *p* = 0.5505) (Figure 4B), as determined by two-sample *t-*test or two-tailed Wilcoxon signed rank test.

### Dexamethasone did not affect the neocortical mRNA expression of EAATs

**Figure 5:**
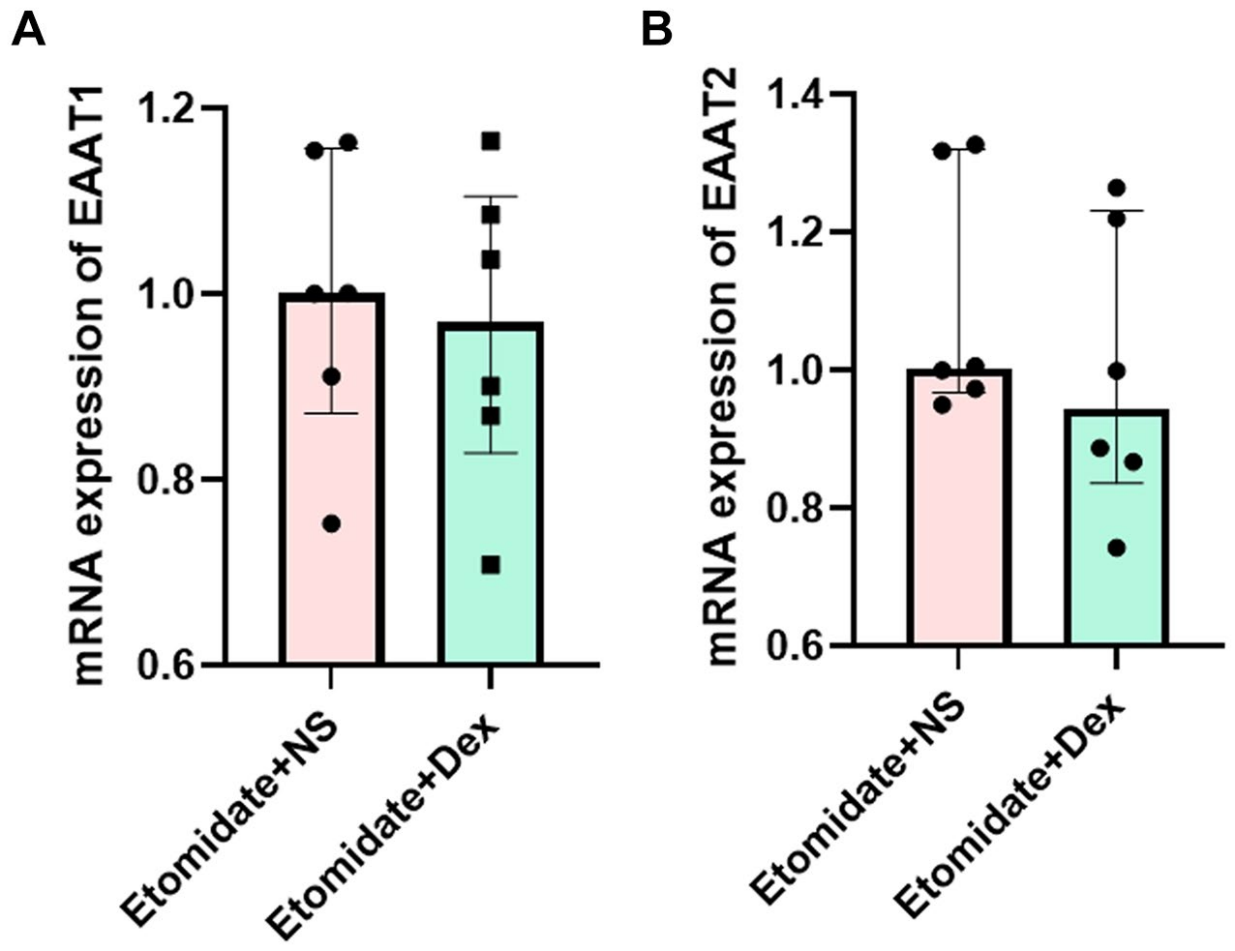
Dexamethasone did not affect the mRNA expression of EAATs in neocortex. **(A)** Expression of EAAT1 mRNA in neocortex in the etomidate + NS group and etomidate + dexamethasone group (*n* = 6 rats/group). **(B)** Expression of EAAT2 mRNA in neocortex in the etomidate + NS group and etomidate + dexamethasone group. A two-tailed *t-*test or two-tailed Wilcoxon signed rank test was applied to analyze difference. Dex, dexamethasone; NS, normal saline.

Treatment with etomidate plus dexamethasone did not affect the mRNA expression of EAAT1 (0.96 ± 0.17 vs. 0.99 ± 0.15, *n* = 6, *p* = 0.7059) (Figure 5A) or EAAT2 (0.99 ± 0.21 vs. 1.10 ± 0.18, *n* = 6, *p* = 0.2403) (Figure 5B) in the neocortex when compared to treatment with etomidate plus NS, as determined by two-sample *t-*test or two-tailed Wilcoxon signed rank test.

### Dexamethasone reversed the etomidate-inhibited protein expression of EAATs

**Figure 6:**
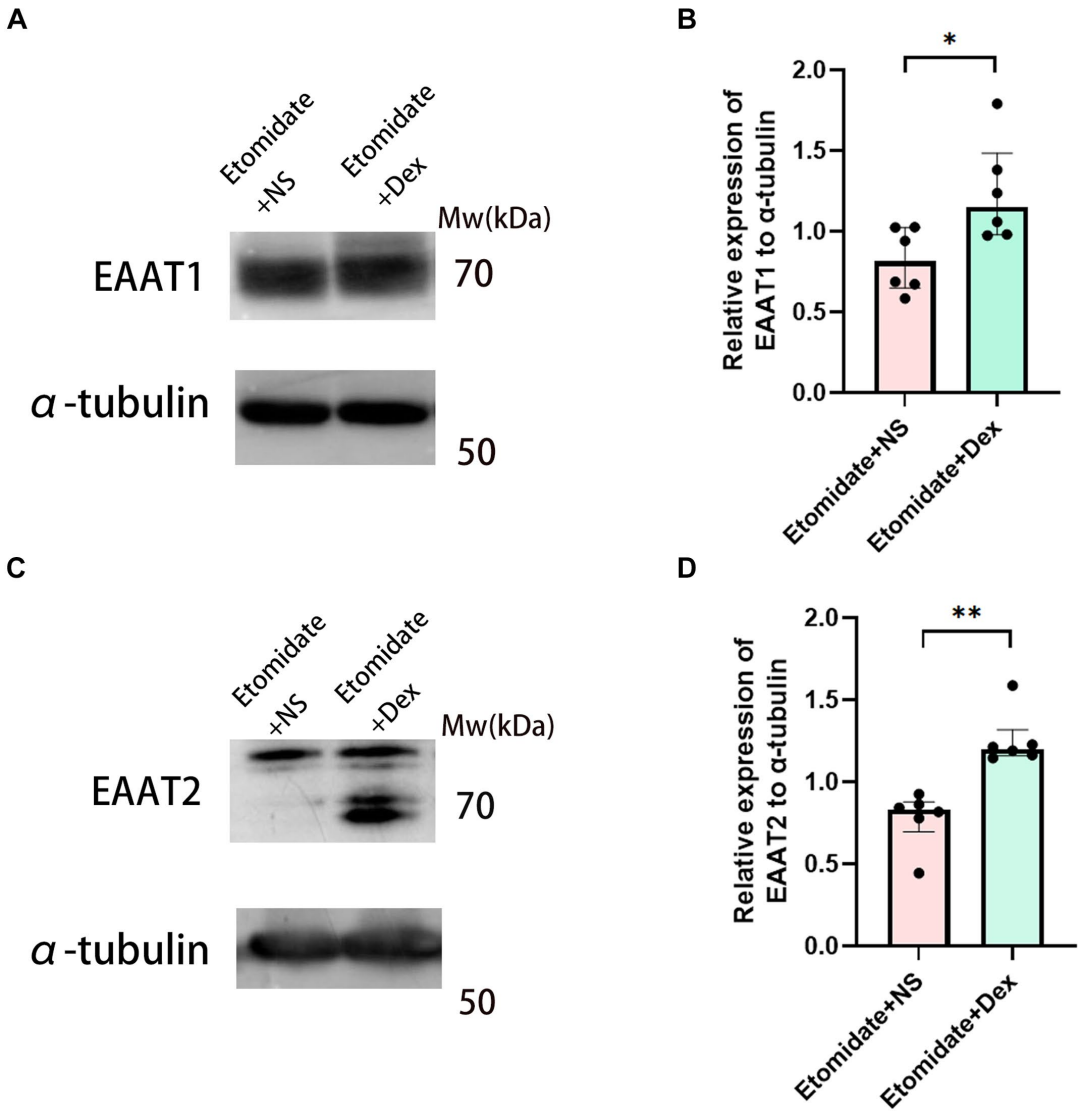
Dexamethasone reversed the inhibition of EAATs in neocortex. Western blot analysis for EAAT1 **(A)** and EAAT2 **(C)** proteins in neocortex in the etomidate + NS group and etomidate + dexamethasone group (*n* = 6 rats/group). Quantification of blot after normalization to α-tubulin of EAAT1 **(B)** and EAAT2 **(D)** in the etomidate + NS group and etomidate + dexamethasone group. ^*^p < 0.05; ^**^p < 0.01, by a two-tailed paired *t-*test or two-tailed Wilcoxon signed rank test. Dex, dexamethasone; NS, normal saline.

In our previous study, etomidate decreased the expression of the EAAT1 and EAAT2 proteins compared to the anticonvulsant doses of propofol and lidocaine (Feng et al., 2022). Intravenous dexamethasone at the end of etomidate was given to explore the effects of dexamethasone on the etomidate-induced myoclonus. We found that etomidate plus dexamethasone increased the expression of EAAT1 (1.24 ± 0.31 vs. 0.82 ± 0.19, *n* = 6, *p* = 0.0207) (Figures 6A,B) and EAAT2 (1.26 ± 0.17 vs. 0.78 ± 0.17, *n* = 6, *p* = 0.0022) (Figures 6C,D) proteins in the neocortex when compared to those in the etomidate plus NS group, as determined by two-sample *t-*test.

### Dexamethasone aggravated the etomidate-inhibited corticosterone

**Figure 7:**
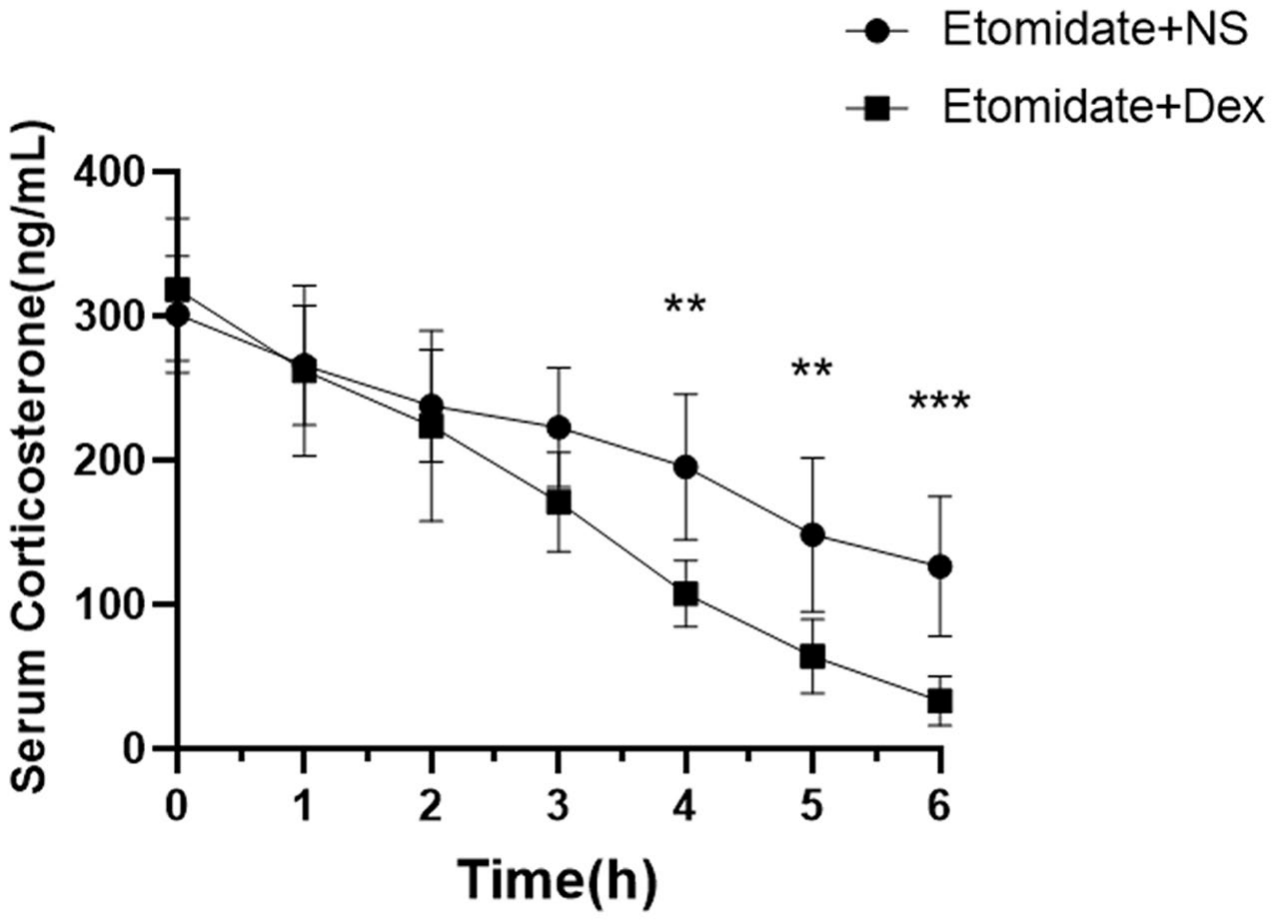
The concentration of serum corticosterone after treatment with etomidate + NS or etomidate + dexamethasone at different time points. ^**^p < 0.01, ^***^p < 0.001, by a linear mixed-model analysis for repeated-measures and a *post hoc* Bonferroni correction test for multiple comparisons. Dex, dexamethasone; NS, normal saline.

The concentrations of serum corticosterone in adult rats are presented in Figure 7. The concentrations of corticosterone were suppressed by etomidate plus NS and etomidate plus dexamethasone over time. Treatment with etomidate plus NS decreased the concentration of serum corticosterone at 2 h (237.80 ± 39.16 vs. 301.20 ± 40.33 ng/mL, *p* = 0.0258), 3 h (222.90 ± 41.51 vs. 301.20 ± 40.33 ng/mL, *p* = 0.0063), 4 h (195.40 ± 50.56 vs. 301.20 ± 40.33 ng/mL, *p* = 0.0010), 5 h (148.60 ± 53.35 vs. 301.20 ± 40.33 ng/mL, *p* = 0.0003), 6 h (126.40 ± 48.58 vs. 301.20 ± 40.33 ng/mL, *p* < 0.0001) after administration compared to the baseline. Etomidate plus dexamethasone decreased the concentration of serum corticosterone at 1 h (262.10 ± 59.08 vs. 318.60 ± 49.56 ng/mL, *p* = 0.0118), 2 h (223.80 ± 65.93 vs. 318.60 ± 49.56 ng/mL, *p* = 0.0014), 3 h (171.20 ± 34.42 vs. 318.60 ± 49.56 ng/mL, *p* < 0.0001), 4 h (107.50 ± 23.13 vs. 318.60 ± 49.56 ng/mL, *p* < 0.0001), 5 h (64.07 ± 25.86 vs. 318.60 ± 49.56 ng/mL, *p* < 0.0001), 6 h (33.23 ± 17.14 vs. 318.60 ± 49.56 ng/mL, *p* < 0.0001) after administration compared to the baseline, as determined by one-way analysis of variance (ANOVA). Etomidate plus dexamethasone decreased the concentration of serum corticosterone at 4 h (*p* = 0.0019), 5 h (*p* = 0.0041), 6 h (*p* = 0.0009) after administration compared to that of etomidate plus NS, as determined by a linear mixed-model analysis for repeated-measures, and a *post hoc* Bonferroni correction test was performed to adjust *p-*values for multiple comparisons.

## Discussion

Etomidate-induced myoclonus has become a clinical safety challenge that has attracted much attention from anesthesiologists. Both etomidate and etomidate analogs have a high incidence of myoclonus, and it has been suggested that the imidazole ring is essential for maintaining the anesthetic effect and inducing myoclonus (Wang et al., 2017). In addition, it is difficult to modify the complication of myoclonus by improving the structure of etomidate, such as phenyl-ring substituted etomidate analogs (McGrath et al., 2019) and ester side-chain substituted etomidate analogs (Wang et al., 2017). As a result, many researchers are searching for the ideal drug for the intervention of etomidate-induced myoclonus. Various drugs (Zhu et al., 2017; Rautela et al., 2023; Shan et al., 2023) have been demonstrated to suppress this myoclonus, including midazolam, fentanyl, remifentanil, magnesium sulfate, lignocaine, dezocine, butorphanol, dexmedetomidine, thiopental, low-dose nalmefene and subhypnotic doses of etomidate. Nevertheless, these drugs can cause adverse effects such as haemodynamic fluctuations, respiratory depression, nausea and vomiting. Research on drug interventions without significant side effects is urgently needed. Dexamethasone not only has a strong anti-inflammatory effect, but also has neuroprotective effects by inhibiting neuroinflammation, an important factor in neuronal damage and microglial activation (Soltani et al., 2023).

In our study, a potential anti-epileptic dose of dexamethasone (4 mg/kg) was chosen according to the previous studies (Sinha et al., 2021) and preliminary experiments revealed that this dose could alleviate the myoclonus and significantly reduce the peak PSD of neocortical ripple waves. This findings verified the neuroprotective effects of dexamethasone and the origination of etomidate-induced myoclonus in the neocortex. An imbalance between glutamate and GABA was the main mechanism of etomidate-induced myoclonus. We found that dexamethasone inhibited etomidate-induced glutamate accumulation during myoclonus. Additionally, although dexamethasone did not affect the neocortical mRNA expression of EAATs during etomidate anesthesia, dexamethasone reversed the etomidate-inhibited the expression of EAAT1 and EAAT2 proteins. In agreement with previous reports, Spang et al. (2017) demonstrated that dexamethasone may lead to a reduction of glutamate production in tendon cells in response to strain *in vitro*. Furthermore, Wen et al. (2005) found that dexamethasone/morphine co-infusion inhibited morphine-evoked glutamate increase and prevented the downregulation of glial glutamate transporters (EAAT1) and (EAAT2). In addition, Zschocke et al. (2005) reported that dexamethasone induced a marked increase in EAAT2 transcription and protein levels, but did not affect EAAT1 transcription and protein expression in cortical astrocytes *in vitro*. The different results in EAAT1 expression induced by dexamethasone in our study may be attributed to the fact that the neocortical tissue was collected from rats *in vivo*, and the inconsistency in EAATs gene and protein expression may be due to the differential location and timing of gene and protein expression under the action of different drugs (Odeon et al., 2015).

Glucocorticoid, which is synthesized in the adrenal gland and secreted into circulation, can penetrate the blood brain barrier and influence neuronal development and plasticity (Taylor and Kokiko-Cochran, 2024). It can activate glucocorticoid receptors (GRs) that widely expressed in the brain (Zschocke et al., 2005). The mechanisms of dexamethasone in reversing the etomidate -inhibited the expression of EAAT1 and EAAT2 proteins may be attributed that dexamethasone activates the GRs, reduces the TrkB-GR interaction and suppresses the BDNF-mediated neurotransmitter release via glutamate transporters (Numakawa et al., 2009). Further researches will be needed to explore the mechanisms of dexamethasone on preventing the above phenomenon.

It is well known that etomidate can cause adrenal suppression at typical anesthetic induction doses in the animals and humans (Pejo et al., 2016; Deng et al., 2020); adrenal suppression seems likely to induce worse outcomes and can be life-threatening in severe cases. Glucocorticoid can induce AI, the impact of dexamethasone on the function of the hypothalamic- pituitary–adrenal (HPA) axis may also be dose dependent (Tang et al., 2019). At present, the effects of dexamethasone combined with etomidate on adrenocortical function in animals are rarely reported. In our study, dexamethasone was administered after etomidate application to evaluate its effects on myoclonus and adrenal suppression. Dexamethasone can attenuate etomidate-induced myoclonus, while conversely aggravating etomidate-induced adrenal suppression. This phenomenon may be related to the fact that glucocorticoids enter the systemic circulation directly and exert negative feedback on corticotropin releasing hormone (CRH)-producing neurons and pituitary corticotroph cells, causing exogenous glucocorticoid suppression of the HPA axis (Tang et al., 2019).

The effects of dexamethasone combined with etomidate on adrenocortical function in patients remain controversial. Dexamethasone administration after etomidate could not only remain normal serum cortisol levels for the duration of the study and follow-up (Levy et al., 1995), but also failed to reduce in-hospital mortality or cardiovascular morbidity after non-cardiac surgery (Komatsu et al., 2018). Etomidate combined with dexamethasone which applied in elderly patients significantly enhanced the duration and severity of adrenal suppression, when compared with etomidate alone or dexamethasone alone (Ting et al., 2022). Careful consideration should be given to the administration of dexamethasone following etomidate anesthesia. Collectively, further studies are needed to verify the effects of the two drugs on adrenocortical function and outcomes of patients.

Our study has several limitations. First, we did not investigate the effects of dexamethasone on astrocyte function following myoclonus. Dexamethasone-mediated enhancement of glutamate transporter function in astrocytes varies in different brain regions (Zschocke et al., 2005). The effect of dexamethasone on astrocyte function following etomidate-induced myoclonus needs further investigation. Second, whether the worsening effect on corticosterone varies with the different concentration of dexamethasone remains uncertain. Third, whether glucocorticoid receptors (GR) can modulate etomidate-induced myoclonus remains unclear. Given that dexamethasone can activate GRs, Zschocke et al. (2005) also found that the enhancing effect of dexamethasone on EAAT2 gene expression and function could be abolished by the GR antagonist mifepristone. Further studies are required. Four, *n* = 6/group for the qRT-PCR and western blot analysis in the study with effect size of 0.98 according to the previous research for the analysis of two-tailed Wilcoxon signed rank test. The study could benefit from a larger sample size to strengthen the statistical significance of the findings.

In conclusion, dexamethasone can alleviate the myoclonus, inhibit the glutamate accumulation, and reverse the etomidate-induced suppression of EAATs in the neocortex, while conversely aggravate etomidate-induced adrenal suppression.

## Data availability statement

The raw data supporting the conclusions of this article will be made available by the authors, without undue reservation.

## Ethics statement

The animal studies were approved by the Animal Ethics Committee of West China Hospital, Sichuan University. The studies were conducted in accordance with the local legislation and institutional requirements. Written informed consent was obtained from the owners for the participation of their animals in this study.

## Author contributions

YF: Data curation, Formal analysis, Methodology, Resources, Supervision, Validation, Visualization, Writing – original draft, Writing – review & editing. MZ: Data curation, Investigation, Writing – original draft. S-yJ: Investigation, Methodology. Y-xG: Conceptualization, Supervision. XJ: Investigation, Writing – review & editing.
